# A Practical Approach to the Management of Residual Cardiovascular Risk: United Arab Emirates Expert Consensus Panel on the Evidence for Icosapent Ethyl and Omega-3 Fatty Acids

**DOI:** 10.1007/s10557-023-07519-z

**Published:** 2024-02-16

**Authors:** Hani Sabbour, Deepak L. Bhatt, Yaser Elhenawi, Asma Aljaberi, Layal Bennani, Tarek Fiad, Khwaja Hasan, Shahrukh Hashmani, Rabih A. Hijazi, Zafar Khan, Ronney Shantouf

**Affiliations:** 1 Warren Alpert School of Medicine, Brown University, RI USA, Mediclinic Hospital, Abu Dhabi, United Arab Emirates; 2 Mount Sinai Fuster Heart Hospital, Icahn School of Medicine at Mount Sinai, New York, NY USA; 3 Heart And Vascular Institute, Cleveland Clinic, Abu Dhabi, United Arab Emirates; 4 Endocrine Division, Department of Medicine, Tawam Hospital, Abu Dhabi, United Arab Emirates; 5 Medical Affairs, Biologix, Dubai, United Arab Emirates; 6 Centre Abu Dhabi, Sheikh Khalifa Medical City, Abu Dhabi, United Arab Emirates; 7 Packer Hospital Guthrie, Sayre, Pennsylvania USA; 8 Department of Endocrinology, Diabetes and Metabolism, Cleveland Clinic Abu Dhabi, Abu Dhabi, United Arab Emirates; 9 Department of Cardiology, Sheikh Shakhbout Medical City, Abu Dhabi, United Arab Emirates

**Keywords:** Hypertriglyceridemia, Dyslipidemia, Residual cardiovascular risk, Omega-3 polyunsaturated fatty acids, Icosapent ethyl, Eicosapentaenoic acid

## Abstract

**Purpose:**

Patients with hyperlipidemia treated with statins remain at a residual cardiovascular (CV) risk. Omega-3 polyunsaturated fatty acids hold the potential to mitigate the residual CV risk in statin-treated patients, with persistently elevated triglyceride (TG) levels.

**Method:**

We reviewed the current evidence on the use of icosapent ethyl (IPE), an omega-3 fatty acid yielding a pure form of eicosapentaenoic acid.

**Results:**

REDUCE-IT reported a significant 25% reduction in CV events, including the need for coronary revascularization, the risk of fatal/nonfatal myocardial infarction, stroke, hospitalization for unstable angina, and CV death in patients on IPE, unseen with other omega-3 fatty acids treatments. IPE was effective in all patients regardless of baseline CV risk enhancers (TG levels, type-2 diabetes status, weight status, prior revascularization, or renal function). Adverse events (atrial fibrillation/flutter) related to IPE have occurred mostly in patients with prior atrial fibrillation. Yet, the net clinical benefit largely exceeded potential risks. The combination with other omega-3 polyunsaturated fatty acids, in particular DHA, eliminated the effect of EPA alone, as reported in the STRENGTH and OMEMI trials. Adding IPE to statin treatment seems to be cost-effective, especially in the context of secondary prevention of CVD, decreasing CV event frequency and subsequently the use of healthcare resources.

**Conclusion:**

Importantly, IPE has been endorsed by 20 international medical societies as a statin add-on treatment in patients with dyslipidemia and high CV risk. Robust medical evidence supports IPE as a pillar in the management of dyslipidemia.

## Introduction

While the cardiovascular (CV) risk incurred from long-standing dyslipidemia, hypertension, diabetes, and other conditions is firmly established, residual CV risk has only recently become of interest in the race to prevent or mitigate CV diseases (CVD). Residual CV risk refers to the risk of emergent CV events that lingers in patients offered standard medical care or in patients with CV risk factors [1, 2]. The concept of residual CV risk stems from clinical trials on lipid-lowering strategies, mainly statin treatments, and extends to the treatment of hypertension, diabetes, and other CV risk factors [3]. Recent evidence supports the initiation of high intensity lipid-lowering agents with ongoing statin therapy [4–6], in an effort to promote further lowering of low-density lipoprotein (LDL)-cholesterol, as well as other atherogenic lipoproteins, and to normalize triglyceride (TG) levels. In fact, the cardio-metabolic risk is based on the concept of risk continuum and must be managed in a holistic manner.

A panel of 10 physicians from the United Arab Emirates (UAE) and the United States of America (USA) convened to review available evidence on the use of omega-3 polyunsaturated fatty acids, and in particular icosapent ethyl (IPE), in the treatment of dyslipidemia and CV risk. Physicians were cardiologists and endocrinologists with expertise in the field of dyslipidemia management and CV protection. Opinions and clinical practice were exchanged in a structured discussion orchestrated by the main author. Recommendations were collected and disseminated among experts before consolidation in the current manuscript.

This manuscript summarizes the state-of-the-art evidence and includes the most recent literature about the use of IPE to mitigate residual CV risk and halt CVD progression. Between June 2021 and June 2023, several drafts were generated to discuss and include newly published papers. This work is a critical appraisal of IPE use in the clinic with a clear comparison with other forms of polyunsaturated fatty acids. The manuscript can serve as a complete compilation of data of IPE and evidence-based guidance for the management of (residual) CV risk.

## Risk Factors and Risk Enhancers in Cardiovascular Disease

### Are Patients with Atherosclerotic CVD, Hypertension, Kidney Disease, Diabetes, or Hyperlipidemia at Higher Risk for CVD?

The combination of specific comorbidities exacerbates CV risk, especially the clustering of largely modifiable risk factors. Abdominal obesity is a major modifiable risk factor tightly associated with insulin resistance, low HDL-cholesterol levels, high TG levels and inflammatory markers, which is usually referred to as atherogenic dyslipidemia. In patients with diabetes, the frequency of CV events was attenuated by 6% upon a decrease of 4 mmHg in systolic blood pressure, by 4% upon a 1 mmol/l (38.7 mg/dl) decrease in LDL-cholesterol and by 1.5% upon lowering of HbA1c by 0.9% [7]. There is also a quantitative association between kidney function (estimated glomerular filtration rate [eGFR]) and the risk of CVD; lower eGFR exponentially increases the CV risk [8, 9]. A direct linear relationship between hyperlipidemia, in particular elevated LDL-cholesterol levels, and the occurrence of CVD, has been well established [10, 11]. Importantly, cardiac events can occur in people with controlled LDL-cholesterol levels (between 2.8 and 3.4 mmol/l or 110 and 130 mg/dl) [12], which calls into question the definition of a “normal” LDL-cholesterol level. Epidemiological data from major statin clinical trials concluded that, despite LDL-cholesterol control, a 56% to 85% residual CV risk remains [13–22] in patients with hypertriglyceridemia [23]. In fact, CV risk amplifies with increasing TG levels up to 1.7 mmol/l (150 mg/dl) in patients with statin-controlled low LDL-cholesterol levels [18, 24]. Collectively, these conditions consort to exacerbate the CV risk and, even when treated, still entail a residual risk [12]. Eradicating CVD in the future warrants lifestyle changes and the use of emerging pharmacotherapies [25].

## Guideline Definitions of CV Risk Categories

### According to International Recommendations, How Do the Different Comorbidities Define CV Risk?

**Fig. 1:**
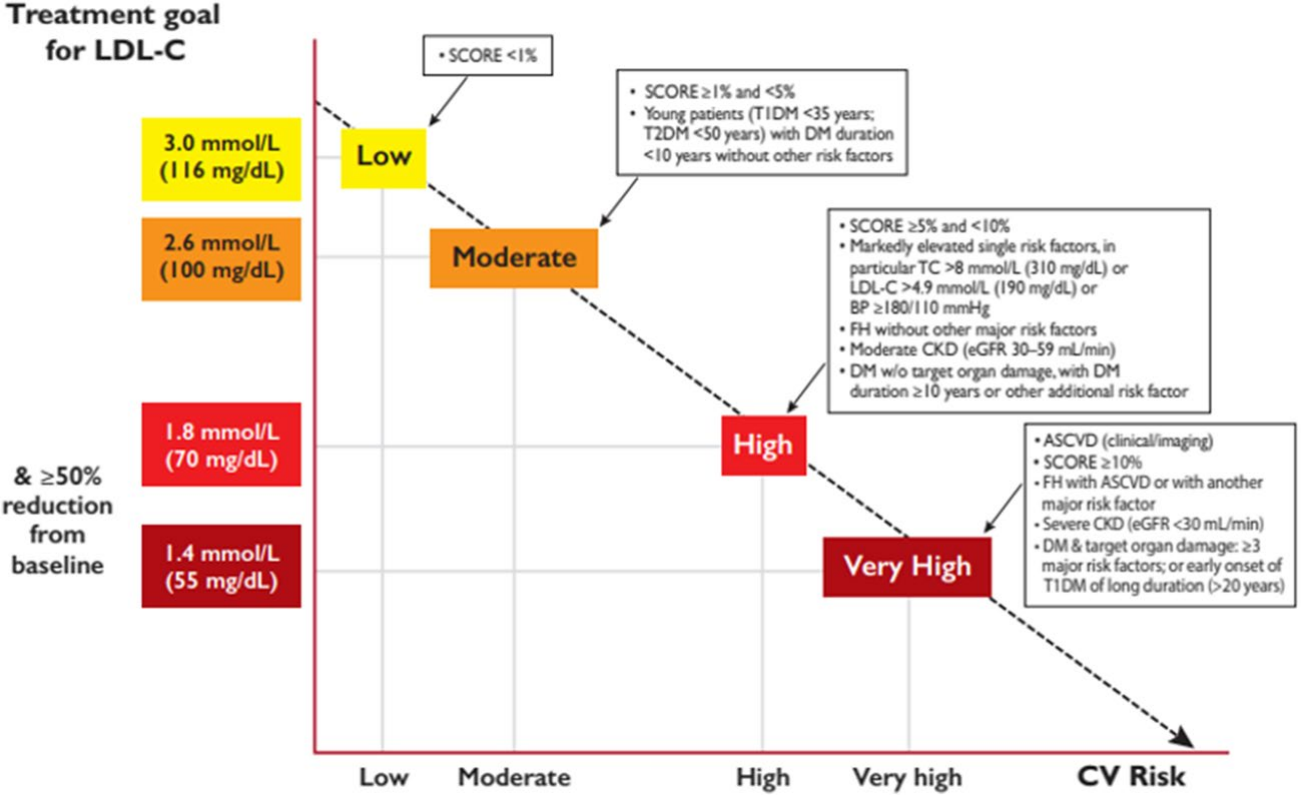
Treatment goals for LDL-cholesterol across categories of total CV risk. ASCVD: atherosclerotic cardiovascular disease; BP, blood pressure; CKD, chronic kidney disease; CV, cardiovascular; DM, diabetes mellitus; eGFR, estimated glomerular filtration rate; FH, familial hypercholesterolemia; LDL-C, low-density lipoprotein cholesterol; SCORE, Systematic Coronary Risk Estimation; T1DM, type-1 DM; T2DM, type-2 DM; TC, total cholesterol [28]. This image is reproduced from the European Heart Journal, Mach et al. [28], upon permission granted by Oxford University Press on behalf of the authors. Not to be reproduced without prior permission from Oxford University Press

Risk categories have been defined and updated, and proposed CV risk scoring systems have been validated or refuted in different populations [26, 27], but some risk factors put the patient at undeniably high risk for CVD. The European Society of Cardiology/European Atherosclerosis Society have defined LDL-cholesterol treatment goals according to different categories of total CV risk [27], as depicted in Fig. 1.

### What is the Standard of Care for Atherosclerotic CVD, Familial Hyperlipidemia, Hypertension, Kidney Disease, and Type II Diabetes, and How are these Conditions Prioritized?

The holistic management of all risk factors is recommended [27]. Statin therapy is recommended in all patients with established CVD, in patients with type-2 diabetes [29], and especially those with chronic kidney disease [30–32]. Antiplatelet therapy is essential in all patients with established CVD [33]. The presence of diabetes mellitus clearly steers the decision toward treating patients with comorbid conditions, such as hypertension, dyslipidemia, or advanced age. However, particularly patients with dyslipidemia and diabetes should be prescribed statins to reduce the risk of coronary heart disease and stroke [34, 35]. Patients with diabetes benefited from intensive statin therapy [36]. Primary prevention is essential to prevent CV events in the broader population based on careful assessment of risk factors, secondary prevention is key to impeding or delaying the onset of recurrent CVD events and complications.

## Triglycerides as a Causal Risk Factor for CVD

### What Blood Lipid Profile is Achieved by Long-Term Statin Treatment?

Highly robust evidence of the CV risk reduction upon long-term statin use [37] makes statins the drugs of choice in the treatment of dyslipidemia and the prevention of atherosclerotic CVD [12, 38], with a rather positive risk/benefit ratio even with the lowest LDL-cholesterol achieved on therapy [39]. Several studies suggest that lowering LDL-cholesterol results in a linear decline in major adverse CV events (MACE); however, all atherogenic components interact to predict residual CVD particularly despite statin therapy [40, 41]. This implies that, while lowering LDL-cholesterol remains at the forefront of dyslipidemia treatment, TG levels should also be monitored as they are considered as a risk enhancer.

### Elevated Triglyceride Levels, Chronic Inflammation, and CVD: Is There an Undeniable Causality?

Epidemiological studies have shown that hypercholesterolemia and advancing age are both risk factors of atherosclerotic CVD; i.e., the greater burden of hypercholesterolemia either due to higher levels or earlier (longer duration) exposure, the earlier the onset of atherosclerotic CVD [42]. Worldwide TG levels are on the rise in different populations, and TG-rich lipoproteins (mostly apolipoprotein [Apo] C3) constitute a CV risk factor, even in the case of elevated HDL-cholesterol and ApoA1 [43]. A recent Mendelian randomization study showed that the reverse is true; LDL-cholesterol or TG-lowering genetic variants are associated with a similar reduction in CVD risk, potentially through ApoB, a common carrier between TG and LDL-cholesterol in the blood [44]. Patients who are not genetically protected against elevated TG or LDL-cholesterol levels would greatly benefit from early treatment initiation to reduce complications from hyperlipidemia. In fact, lowering LDL-cholesterol by 0.025 mmol/l (4.9 mg/dl) and TG by 0.27 mmol/l (24 mg/dl) has been shown to result in a substantial reduction in CV events. However, statins, despite decreasing LDL-cholesterol levels (sometimes aggressively to below 1.8 mmol/l or 70 mg/dl), still do not eradicate CV risk. This, coupled with the persistence of TG and other subsets of cholesterol-carrier lipoproteins, results in the persistence of a residual CV risk [45]. These studies serve to refocus attention on TG and position TG lowering as an important factor, alongside LDL-cholesterol, in the fight against atherosclerotic CVD.

There is a tight interplay between lipid metabolism and chronic inflammation. Atherosclerotic disease, in all its stages, presents with chronic low-grade inflammation [46–48]. Indeed, inflammation, through elevated blood C reactive protein (CRP) levels, is an established CVD risk enhancer [48, 49], as defined by international guidelines. CRP levels decrease upon treatment with statins [47, 50] and with omega-3 fatty acids [51]. The Reduction of Cardiovascular Events with Icosapent Ethyl–Intervention Trial (REDUCE-IT) showed that high-sensitivity CRP (hsCRP) level was reduced by 39.9% as compared to placebo, which was statistically significant in patients who already achieved target LDL-cholesterol levels on statin therapy [52], in line with the literature [53]. Addition of IPE treatment to statin resulted in a reduction in major CV events in primary and secondary prevention settings, mainly CV death, nonfatal myocardial infarction, nonfatal stroke, coronary revascularization [54] as well as significant improvement of plaque regression [55–57].

## Residual CV Risk: Definition, Pharmacological Management

### How is Residual CV Risk Evaluated in Clinical Trials (Biomarkers Versus Clinical Manifestations)?

Residual CV risk is defined as an extremely high risk of CV events in patients already treated for CV risk factors or recurring/progressive atherosclerotic CVD [58], which might occur in other arterial territories (cerebral vascular accident, CVD, peripheral artery disease). Residual risk clinically manifests itself by the occurrence of a new or a repeated CV event in spite of achieving target LDL-cholesterol (< 1.8 mmol/l or 70 mg/dl), suggesting that the current therapeutic approach has failed to prevent onset or progression of CVD. This is driven by a plethora of factors, including biochemical markers (high TG and low HDL levels, elevated levels of lipoprotein(a), apolipoprotein B and CRP), metabolic syndrome, insulin resistance, or established diabetes [58].

### What is the Existing Evidence for Mitigating Residual CV Risk in Statin-Treated Patients?

There is considerable heterogeneity in the outcomes of TG-lowering therapies that differ based on the pharmacological mechanism. As recently reviewed, fibrates have been extensively evaluated. The Veterans Affairs Cooperative Studies Program High-Density Lipoprotein Cholesterol Intervention (1999 VA-HIT) trial, the 2005 Fenofibrate Intervention and Event Lowering in Diabetes (FIELD) trial, and the 2010 Action to Control Cardiovascular Risk in Diabetes (ACCORD)-Lipid trial reported a significant decrease in TG levels upon treatment with gemfibrozil, fenofibrate, and combined statin/fenofibrate, respectively [59]. However, in the FIELD trial, except in the subgroup of patients with elevated TG and low HDL-cholesterol levels, the risk of coronary events (death or nonfatal myocardial infarction) was not mitigated upon fibrate use [60]; and in the ACCORD-Lipid trial, 200 mg fibrates did not lead to reduction in the rate of fatal CV events, nonfatal myocardial infarction, or stroke [61]. The phase 3 Pemafibrate to Reduce Cardiovascular Outcomes by Reducing Triglycerides in Patients with Diabetes (PROMINENT) study planned to investigate the effects of pemafibrate on risk of CV events in high-risk patients with type-2 diabetes, mild-to-moderate hypertriglyceridemia, and low levels of HDL-cholesterol treated with statins [62]. The primary endpoint was a composite of nonfatal MI, nonfatal ischemic stroke, coronary revascularization, and CV death. The PROMINENT study was discontinued early based on the recommendations of the Data Safety Monitoring Board (DSMB). Despite lowering TG levels, pemafibrate did not decrease the incidence of CV events compared to placebo. However, pemafibrate was associated with a higher incidence of adverse renal events and venous thromboembolism despite the similar overall incidence of serious adverse events between groups [63].

Niacin is no longer recommended in the guidelines for the management of hyperlipidemia, driven by the following evidence: TG levels were only marginally decreased upon treatment with niacin alone or in association with laropiprant in the 2011 Atherothrombosis Intervention in Metabolic Syndromes with Low HDL/High Triglycerides and Impact on Global Health Outcomes (AIM-HIGH) trial [64] and the 2014 Heart Protection Study 2-Treatment of HDL to Reduce the Incidence of Vascular Events (HPS2-THRIVE) [65], respectively.

Omega-3 fatty acids have been extensively studied since the original Gruppo Italiano per lo Studio della Sopravvivenza nell’Infarto (GISSI) Prevention study in 1999. In that study, treatment in post-myocardial infarction patients with omega-3 polyunsaturated fatty acids resulted in significant decrease in TG levels and a decreased risk of CV event [66]. The more recent Japan EPA Lipid Intervention Study (JELIS) trial utilizing modern preventive therapy, including statin, highlighted the beneficial effects of eicosapentaenoic acid (EPA) on TG lowering and significant CV risk reduction, even at lower doses. Additionally, the Randomized Trial for Evaluating Secondary Prevention Efficacy of Combination Therapy - Statin and Eicosapentaenoic Acid (RESPECT-EPA) study followed around 3900 patients with stable coronary artery disease, randomized to 1.8 g/day IPE versus no IPE, on top of statin treatment, for the primary endpoint of major adverse cardiac events (cardiovascular death, myocardial infarction, stroke, unstable angina requiring hospitalization, and revascularization) [67–69]. The RESPECT-EPA trial found that the primary outcome occurred in 10.9% of patients on IPE compared to 14.9% of control patients, with a trend toward statistical significance (*P* = 0.055). In addition, the secondary outcomes (sudden cardiac death, myocardial infarction, unstable angina, or coronary revascularization) occurred less frequently in the IPE group than in the control group (8.0% versus 11.3%, *P* = 0.031) [70].

Table 1 revisits the major outcomes of studies on omega-3 fatty acid use in mitigating CV risk.

**Table 1:** Key trials of omega-3 fatty acids that failed to provide CV protection, except for IPE

Agent	Trial	Patients’ characteristics	Study drug	Key findings
Prescription Pure EPA	REDUCE-IT [52, 71]	Western patients, with established CV disease or with diabetes and other risk factors, who had been receiving statin therapy, who had a fasting triglyceride level of 135 to 499 mg/dl	4 g IPE	Met primary endpoint, EPA + statin reduced MACE The risk of the primary composite endpoint was significantly lower by 25%, among patients on IPE versus placebo
	JELIS [54]	Japanese patients with total cholesterol of ≥6.5 mmol/L	1.8 g IPE	Met primary endpoint, EPA + statin reduced MACE 19% relative reduction in MACE among patients on IPE versus statin alone
	RESPECT-EPA [70]	Japanese patients with chronic CAD on Statins	1.8 g IPE	IPE reduces adverse CV outcomes CV events occurred in only 10.9% of the IPE group versus 14.9% of the control group
EPA + DHA mixtures	ORIGIN [72]	Patients at high risk for CV events and had impaired fasting glucose, impaired glucose tolerance, or diabetes	1 g omega-3-acid ethyl esters	No effect on CV outcomes The incidence of the primary outcome was not significantly decreased among patients receiving omega–3 fatty acids (9.3%) versus placebo (9.1%) group
	RISK & PREVENTION [73]	Patients with multiple CV risk factors or atherosclerotic vascular disease but not MI	1 g EPA + DHA mixture	No reduction in CV morbidity and mortality
	OMEGA [74]	Survivors of acute MI	1 g omega-3-acid ethyl esters	No reduction in rate of clinical events
	ASCEND [75]	Patients with diabetes who did not already have any existing problems with their heart or blood circulation	1 g omega-3-acid ethyl esters	No significant difference in the risk of MACE
	VITAL [76]	US adults without cancer or CVD	1 g omega-3-acid ethyl esters	Did not meet primary endpoint
	STRENGTH [37, 77]	Patients with dyslipidemia and high CV risk	4 g omega-3 carboxylic acids	*Study discontinued in 2020* Analysis showed no significant difference in a composite outcome of MACE

*ASCEND*, A Study of Cardiovascular Events in Diabetes; *CV*, cardiovascular; *DHA*, docosahexaenoic acid; *EPA*, eicosapentaenoic acid; *IPE*, icosapent ethyl; *MACE*, major cardiovascular events; *MI*, myocardial infarction; *OMEGA*, Effect of Highly Purified Omega-3 Fatty Acids on Top of Modern Guideline-Adjusted Therapy After Myocardial Infarction; *ORIGIN*, Outcome Reduction With Initial Glargine Intervention; *STRENGTH*, Outcomes Study to Assess STatin Residual Risk Reduction With EpaNova in HiGh CV Risk PatienTs With Hypertriglyceridemia; *VITAL*, Vitamin D and Omega-3 Trial; *US*, United States

###  How Does the Cardio-Protective Role of IPE Unfold? Results of REDUCE-IT 

Several omega-3 fatty acids have been evaluated as TG-lowering agents, showing successful lowering of TG levels. However, only pure IPE was associated with proven and sustained CV benefits. In fact, mixtures of other low dose and high dose omega-3 fatty acids showed no significant CV benefit in terms of coronary heart disease, stroke, revascularization, or any major vascular event [78].

A landmark clinical trial, REDUCE-IT, confirmed the relevance of evaluating medications that further lower TG levels and simultaneously yield different outcomes; i.e., TG level reduction and mitigation of CV events. REDUCE-IT evaluated the effects of IPE on the composite CV outcome (CV death, nonfatal myocardial infarction, nonfatal stroke, coronary revascularization, and unstable angina requiring hospitalization). As shown in Table 2, compared to placebo, the proportion of patients with a CV event occurring in the 5 years following randomization was significantly lower by 25% [52]. Taken separately, CV outcomes were also significantly reduced in patients on IPE compared to those on placebo. A 35% reduction in revascularization was reported in REDUCE-IT, compared with a 24% reduction with stenting in the Norwegian Coronary Stent Trial (NORSTENT) trial, 22% in the Further Cardiovascular Outcomes Research With PCSK9 Inhibition in Subjects With Elevated Risk (FOURIER) trial, and 12% in the Evaluation of Cardiovascular Outcomes After an Acute Coronary Syndrome During Treatment With Alirocumab (ODYSSEY OUTCOMES) trial. The revascularization risk reduction with IPE (35%) was comparable to that reported with simvastatin versus placebo use in 1994 (37%) [79]. In addition to decreasing the number of overall CV events, IPE also resulted in significantly fewer second, third, and fourth events (i.e., continuous reduction of recurrent CV events), compared to placebo [80].

**Table 2:** CV event risk reduction in patients on IPE compared to placebo (from REDUCE-IT)

Endpoint	HR (95% CI)	% Risk reduction	*P* value
Primary composite^a^ (ITT)	0.75 (0.68–0.83)	25%	< 0.001
Key secondary composite^b^ (ITT)	0.74 (0.65–0.83)	26%	< 0.001
CV death or nonfatal MI	0.75 (0.66–0.86)	25%	< 0.001
Fatal or nonfatal MI	0.69 (0.58–0.81)	31%	< 0.001
Urgent or emergent revascularization	0.65 (0.55–0.78)	35%	< 0.001
CV death	0.80 (0.66–0.98)	20%	0.03
Hospitalization for unstable angina	0.68 (0.53–0.87)	32%	0.002
Fatal or nonfatal stroke	0.72 (0.55–0.93)	28%	0.01
Total mortality, nonfatal MI, or nonfatal stroke	0.77 (0.69–0.86)	23%	< 0.001
Total mortality	0.87 (0.74–1.02)	13%	0.09
Sudden cardiac death^c^	0.69 (0.50–0.96)	31%	--
Cardiac arrest^c^	0.52 (0.31–0.86)	48%	--

*CI*, confidence interval; *CV*, cardiovascular; *HR*, hazard ratio; *ITT*, intention to treat; *MI*, myocardial infarction

^a^Primary composite endpoint: CV death, nonfatal MI or stroke, coronary revascularization, or unstable angina.

^b^Key secondary composite endpoint of CV death, nonfatal MI or stroke.

^c^Data from Supplementary Appendix of the work by Bhatt et al. [52]

Importantly, the primary composite outcome was significantly reduced regardless of baseline TG levels whether modestly elevated (> 1.69 mmol/l or 150 mg/dl) or more severely elevated (> 2.26 mmol/l or 200 mg/dl); both groups equally benefited from substantial reduction in CV events with IPE group [80]. This means that TG is a marker of increased risk and the addition of IPE provides a substantial benefit regardless of TG level studied, which has led to the label indication for reduction in CV events for patients with elevated TG (> 1.69 mmol/l or 150 mg/dl).

Compared to placebo, all CV outcomes significantly improved with IPE use throughout the study, and both primary and secondary outcomes were sustained over at least 2 years. In REDUCE-IT, for every 1000 patients treated with IPE and followed over 5 years, 12 fewer CV deaths, 42 fewer myocardial infarctions, 14 fewer strokes, 76 fewer coronary revascularization interventions, 16 fewer hospitalization for unstable angina, and 159 fewer total primary outcome events were reported, compared to placebo-treated patients [80].

**Fig. 2:**
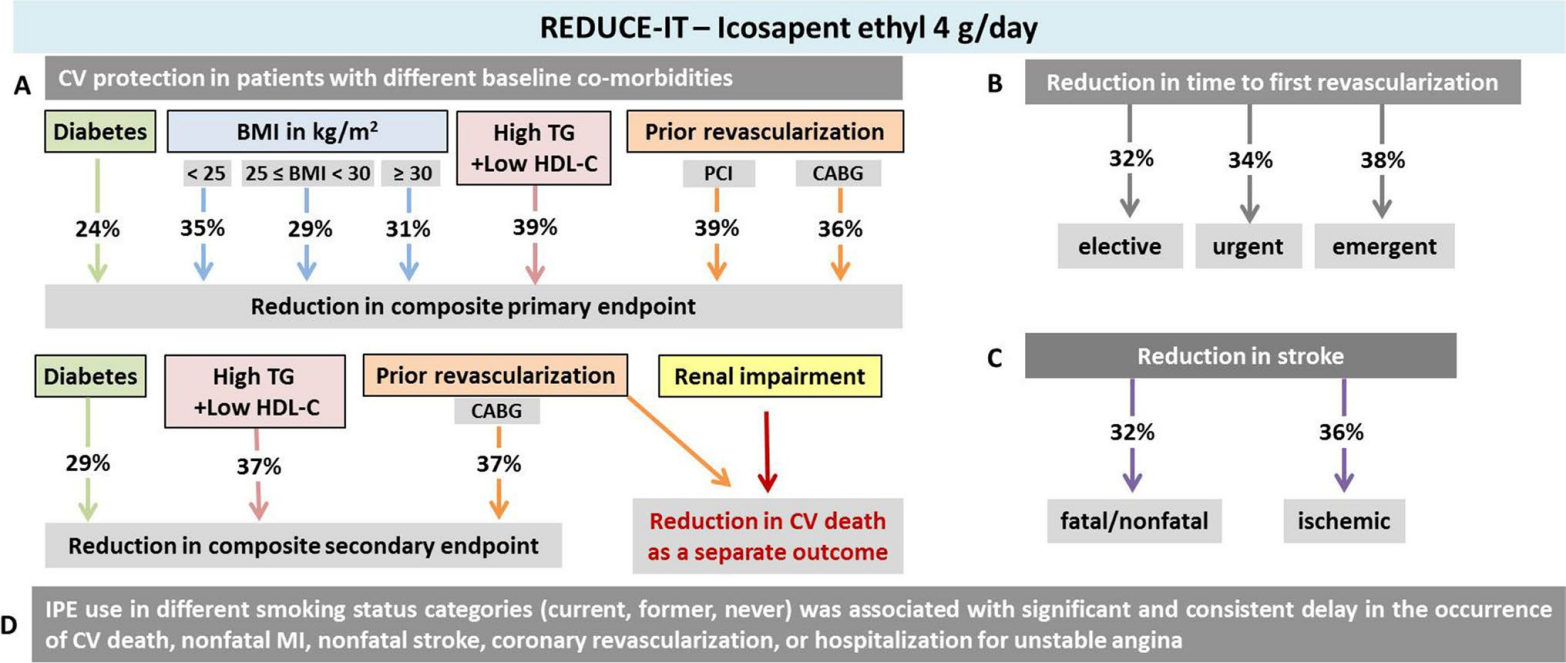
IPE at 4 g daily confers CV benefits in patients with and without hyperlipidemia (from REDUCE-IT). **A**. IPE resulted in a reduction in the rate of total primary and secondary endpoints for patients with different baseline characteristics. **B**. Compared to placebo, there was an increase in time to first revascularization of at least 32% in patients treated with IPE **C**. IPE decreased the incidence of total stroke events. **D**. IPE use was associated with significant and consistent delay in the occurrence of CV events in all smoking status categories (A stand-alone post hoc analysis). *Primary endpoint*: composite of CV death, nonfatal myocardial infarction, nonfatal stroke, coronary revascularization, or unstable angina. *Key secondary endpoints*: a composite of CV death, nonfatal myocardial infarction, or nonfatal stroke. High TG: TG ≥200 mg/dl. Low HDL-C: HDL-C ≤35 mg/dl. CV death was also displayed separately, given its importance as the ultimate benefit. BMI, body mass index; CABG, coronary artery bypass graft; CV, cardiovascular; HDL-C, High-density lipoprotein cholesterol; IPE, icosapent ethyl; MI, myocardial infarction; PCI, percutaneous coronary intervention; TG, triglycerides. [81–87]

 REDUCE-IT was further dissected according to population subgroups. Figure 2 below summarizes the effects of IPE treatment in patients at high CV risk, and with different comorbid conditions [81–87]. Recently, the effect of IPE was evaluated in post-hoc analyses of REDUCE-IT, examining the benefits of IPE in patients with different smoking history (current, former, and never smokers). The time to primary composite endpoint (CV death, nonfatal myocardial infarction, nonfatal stroke, coronary revascularization, or hospitalization for unstable angina) was once again greatly prolonged in IPE-treated patients compared to placebo, regardless of smoking status [88].

### Could the Mechanism of Action of IPE Explain the Substantial Magnitude of the Positive Outcomes with IPE Compared to Other Omega-3 Fatty Acids?

**Fig. 3:**
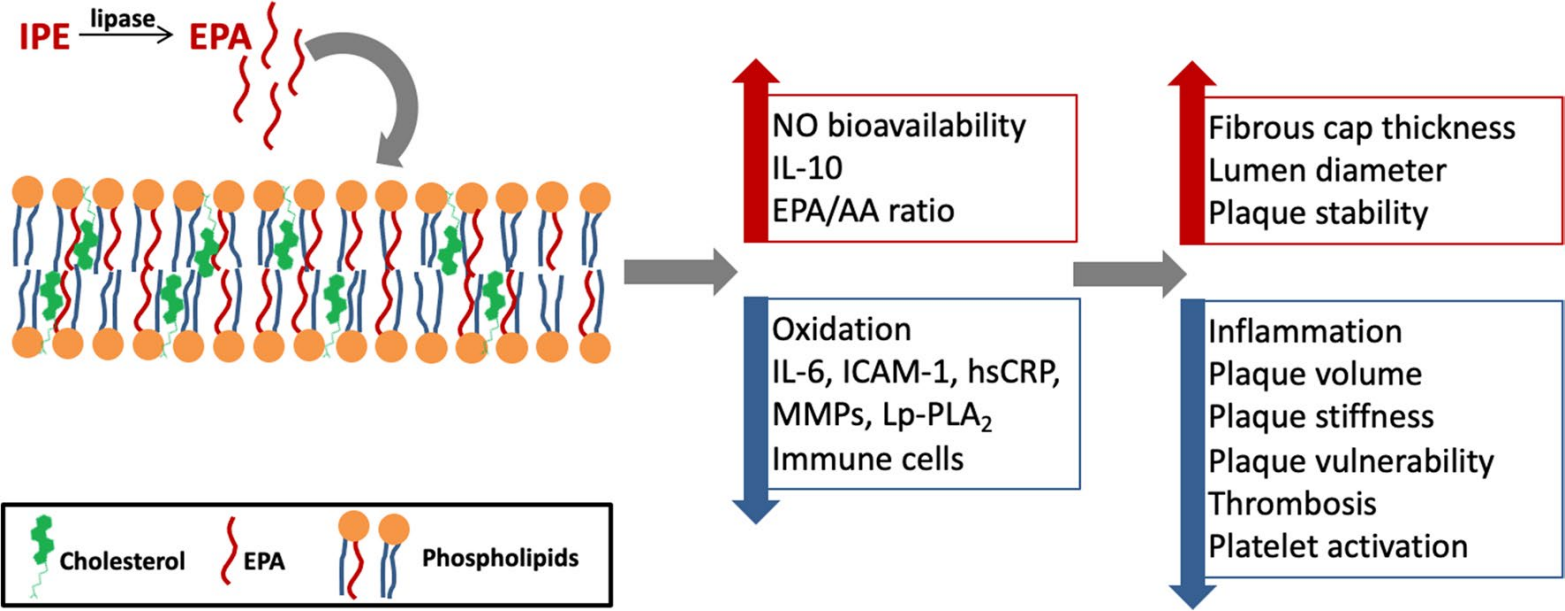
Mechanisms of action of IPE, driving its effects on atherosclerotic plaque.  EPA incorporates in the phospholipid bilayer, promoting anti-inflammatory processes, decreasing lipid oxidation, immune cell recruitment, and pro-inflammatory processes [45, 57]. Preclinical in vitro studies report that EPA reduces the expression of pulmonary ACE and ICAM-1, upregulated in response to pro-inflammatory IL-6, preserving vascular endothelial function [90, 91]. AA, arachidonic acid; ACE, angiotensin-converting enzyme; EPA, eicosapentaenoic acid; hsCRP, high sensitivity C reactive protein; ICAM-1, intercellular adhesion molecule 1; IL, interleukin; IPE, icosapent ethyl; Lp-PLA2, lipoprotein-associated phospholipase A2; MMPs, matrix metalloproteinases; NO, nitric oxide

IPE is a substrate for intestinal lipase, which de-esterifies it to yield EPA. EPA diffuses into the intestinal epithelial cells, where it is re-esterified and packaged into chylomicrons that cross into the lacteals, to eventually join the circulation [89]. EPA incorporates in the lipid bilayer of the plasma membrane, without disrupting its architecture, cholesterol distribution or normal fluidity. Figure 3 summarizes the molecular effects of IPE, which yield its CV effects through potential antioxidant properties and promoting anti-inflammatory processes. Conversely, docosahexaenoic acid (DHA), another major marine-derived omega-3 fatty acid, which also incorporates in the plasma membrane, increases its fluidity and modulates lipid domains, with reduced antioxidant activity [90].

In contrast to other omega-3 trials with specific omega-3 formulations, the magnitude of benefits in CV risk seen in REDUCE-IT has not been replicated. It can be concluded that IPE is the specific agent behind this CV benefit that was not reported in the STRENGTH trial using omega-3 carboxylic acid combination.

The STRENGTH trial of a specific omega-3 carboxylic acid composition (EPA + DHA) did not lead to improved CV outcomes, compared to placebo and was discontinued prematurely [92]. In the Omega-3 Fatty acids in Elderly with Myocardial Infarction (OMEMI) trial that was a smaller study, administering a mixture of EPA and DHA to elderly patients’ post-myocardial infarction did not improve CV outcomes compared to placebo [93]. Findings from REDUCE-IT , as well as from other trials evaluating omega-3 fatty acids, were scrutinized in an attempt to identify the reason behind discrepancy in CV outcomes upon treatment with the different omega-3 fatty acid agents. From a pharmacological standpoint, this would appear to be a comparison between pure IPE therapies versus therapies that contain a mixture of EPA and DHA.

### Could the Use of Mineral Oil in the Placebo Arm Explain the Magnitude of Benefits?

Some have argued that the substantially positive results from REDUCE-IT might be attributed to a negative CV effects of the mineral oil-based placebo rather than the beneficial effects of IPE [94]. The safety of pharmaceutical grade mineral oil was investigated and its use as placebo in clinical trials was found to be acceptable and not biasing trial outcomes [95].

Despite robust evidence, controversy has been raised about the interpretation of REDUCE-IT. However, statistical analysis reveals that the effect of IPE cannot be due to chance given the robust study design and power to detect statistical difference. Second, confounding factors, such as the use of mineral oil in the placebo arm has also been rigorously analyzed; only small absolute changes in inflammatory and lipid biomarkers other than TG were found, and these were not associated with CV outcomes in the study [95, 96]. In addition, the 2020 review did not identify any consistent trend in lipid levels or inflammatory markers in patients given mineral oil. Furthermore, an analysis of REDUCE-IT by baseline statin use (hydrophilic versus hydrophobic) found consistent benefits, again arguing against any interaction between mineral oil and statins [97]. This reassurance on the robust conclusions obtained by REDUCE-IT is further backed up by results from the JELIS trial, which did not use mineral oil as placebo [54]. Additionally, very recent research reported that IPE inhibits LDL-cholesterol oxidation, compared to mineral oil and DHA in vitro [98]. In particular, the rate of LDL-cholesterol oxidation was not affected by mineral oil (*P* < 0.001), while EPA significantly inhibited LDL-cholesterol oxidation compared to vehicle (*P* < 0.001). DHA exerted its anti-oxidant activity only for 2 h, and to a lesser level than EPA (*P* < 0.05). The longer-term antioxidant potential of EPA may contribute to decreased incidence of CV events [98]. Another in vitro study demonstrated that the antioxidant activity of mineral or corn oil was inexistent, even at supra-pharmacological doses, underscoring that the antioxidant activity is actually attributable to IPE and not placebo choice [99].

### Does Elevated Serum EPA Level Reduce CV Risk?

Atherosclerotic plaque remodeling has been used for decades as a surrogate for CV outcomes, and a proof for the biological effects of statins (the SATURN trial [100]) and of proprotein convertase subtilisin/kexin type-9 (PCSK9) inhibitors (the GLAGOV trial [101]). The Combination Therapy of Eicosapentaenoic acid and Pitavastatin for Coronary Plaque Regression Evaluated by Integrated Backscatter Intravascular Ultrasonography (CHERRY) trial results support that prescription EPA reduces coronary plaque volume [55]. The more recent Effect of Vascepa on Improving Coronary Atherosclerosis in People with High Triglycerides Taking Statin Therapy (EVAPORATE) trial also confirms that IPE decreases plaque volume by 9% (versus an increase by 11% in the placebo arm) and improves plaque composition [57, 102]. Looking at these mechanistic studies, which report on atherosclerotic plaque regression with IPE, it is abundantly clear that neither study design nor the comparator placebo arm could explain the difference in outcome between combination EPA and DHA treatment or EPA alone, except the beneficial effect of IPE. The 25% reduction in CV risk cannot therefore be attributed to statistical chance or to any theoretical mineral oil contribution. Further scrutiny showed that the advantage of IPE over other omega-3 fatty acids might be conferred by the greater stability of this omega-3 fatty acid that exerts its lipid-lowering effects through EPA. Indeed, achieved serum EPA levels directly correlate with CV protection, regardless of their TG-lowering effects; since TG is a mediator of CVD and lower TG levels do not necessarily imply lower CV risk. EPA is believed to mitigate this CVD risk in patients with moderate or high levels of TG. As per REDUCE-IT, IPE resulted in the highest serum EPA levels and was associated with a 25% CV risk reduction [103], compared to lower serum EPA levels in patients administered other forms of omega-3 fatty acids [92].

### How is IPE Positioned in the International Dyslipidemia Guidelines?

Year 2021 witnessed the endorsement of IPE by many international medical societies. Table 3 lists the international recommendations endorsing the use of IPE, along the road to its approval and up to currently applicable guidelines.

**Table 3:** Recommendations on the use of IPE: the road to approval and beyond

	Statements – *verbatim*
2021	
American Heart Association/American Stroke Association	In patients with ischemic stroke or TIA, with fasting triglycerides 135 to 499 mg/dl and LDL-C of 41 to 100 mg/dl, on moderate- or high-intensity statin therapy, with HbA1c < 10%, and with no history of pancreatitis, AF, or severe heart failure, treatment with IPE 2 g twice a day is reasonable to reduce risk of recurrent stroke [104]. COR: 2a
American College of Cardiology	Adults ≥50 years with one or more ASCVD high-risk feature, and persistent fasting hypertriglyceridemia 150-499 mg/dl may be considered for IPE treatment [105]
European Society of Cardiology	In high-risk (or above) patients with triglycerides >1.5 mmol/l (135 mg/dl) despite statin treatment and lifestyle measures, n-3 PUFAs (IPE 2 x 2 g/day) may be considered in combination with a statin [106]. Class IIb B
Saudi Health Council	Recently IPE (a form of omega 3 not available in KSA [*at the time of writing*]) was shown to reduce CV events including deaths in adults with moderate hypertriglyceridemia (fasting or nonfasting triglycerides 175–499 mg/dl) [107].
2020	
American Diabetes Association	The Standards of Care now include a recommendation that IPE be considered for patients with diabetes and atherosclerotic cardiovascular disease (ASCVD) or other cardiac risk factors on a statin with controlled LDL-C, but with elevated triglycerides (135–499 mg/dl) to reduce cardiovascular risk [108]. *Updates to the 2019 Standards of Medical Care in Diabetes* https://care.diabetesjournals.org/content/45/Supplement_1/S144/138910/10-Cardiovascular-Disease-and-Risk-Management
American Heart Association	Consider IPE for further cardiovascular risk reduction when triglycerides remain elevated (>135 mg/dl) despite maximally tolerated statin [109].
Canadian Stroke Best Practice Recommendations	Add-on therapies for hypertriglyceridemia (New 2020) For ischemic stroke patients with established atherosclerotic cardiovascular disease or diabetes plus additional vascular risk factors, who have elevated serum triglyceride levels (≥1.5 mmol/l) despite statin therapy, IPE 2 g bid may be considered to decrease the risk of vascular events [110] LOE B.
2019	
European Society of Cardiology/ European Atherosclerosis Society	The new guidelines have taken account of evidence from REDUCE-IT and recommend n-3 PUFAs (particularly IPE 2 x 2 g daily) in high-risk patients with persistently elevated TG (between 135–499 mg/dl or 1.5 and 5.6 mmol/l) despite statin treatment [111]. *Update to Dyslipidemia Guidelines 2019:* https://academic.oup.com/eurheartj/article/41/1/111/5556353
National Lipid Association	Patients aged ≥45 years with clinical ASCVD, or aged ≥50 years with diabetes mellitus requiring medication plus ≥1 additional risk factor, with fasting triglycerides 135 to 499 mg/dl on high-intensity or maximally tolerated statin therapy (±ezetimibe), treatment with IPE is recommended for ASCVD risk reduction [112] (class I; LOE: B-R).

*AF*, atrial fibrillation; *ASCVD*, atherosclerotic cardiovascular disease; *COR*, class of recommendation; *HbA1c*, glycated hemoglobin A1; *IPE*, icosapent ethyl; *LDL-C*, low-density lipoprotein-cholesterol; *LOE*, level of evidence; *n-3 PUFA*, omega-3 polyunsaturated fatty acids; *TIA*, transient ischemic attack; *TG*, triglycerides

### Which Patients are Candidates for IPE Treatment?

Patients with high TG levels and controlled LDL-cholesterol levels were enrolled in REDUCE-IT and those in the **IPE** treatment arm showed significantly lower risk for new-onset or recurring CVD.

IPE can be used for primary or secondary prevention purposes. Table 4 lists the risk-enhancing criteria that make patients eligible for IPE treatment, depending on clinical phenotype. Interestingly, there is a robust medical profile that qualifies patients for primary prevention use of IPE.

**Table 4:** Eligibility for IPE use for primary or secondary prevention purposes [71]

Primary prevention	Secondary prevention
Diabetes mellitus requiring medication AND ≥ 50 years of age AND ≥ 1 additional risk factor for CVD • Men ≥ 55 years and women ≥ 65 years • Cigarette smoker or stopped within 3 months • Hypertension (SBP ≥ 140 mmHg OR DBP ≥ 90 mmHg) or on antihypertensive treatment • HDL-C ≤ 1.03 mmol/l (40 mg/dl) for men and ≤ 1.3 mmol/l (50 mg/dl) for women • hsCRP > 3.0 mg/l • Renal dysfunction: creatinine clearance rate between 30 and 60 ml/min • Retinopathy • Micro- or macro-albuminuria • ABI < 0.9 without symptoms of intermittent claudication	One of the following cases must exist Documented CAD • Multi-vessel CAD (≥ 50% stenosis in ≥ 2 major epicardial coronary arteries, with or without antecedent revascularization • Prior MI • Hospitalization for high-risk non-ST-segment elevation, acute coronary syndrome with ST-segment deviation or biomarker positivity Documented cerebrovascular or carotid disease • Prior ischemic stroke • Symptomatic carotid artery disease with ≥ 50% carotid arterial stenosis • Asymptomatic carotid artery disease with ≥ 70% carotid arterial stenosis • History of carotid revascularization Documented PAD • ABI < 0.9 with symptoms of intermittent claudication • History of aorto-iliac or peripheral artery intervention

*ABI*, ankle-brachial index; *CAD*, coronary artery disease; *DBP*, diastolic blood pressure; *HDL-C*, high-density lipoprotein cholesterol; *hsCRP*, high-sensitivity C-reactive protein; MI: myocardial infarction; *PAD*, peripheral artery disease; *SBP*, systolic blood pressure

Patients with dyslipidemia (particularly elevated TG levels), in addition to those with other morbid conditions and with different smoking histories, as shown in Fig. 1, greatly benefit from IPE treatment. While all patients with CV risk factors might benefit from IPE treatment for primary prevention, patients with atherogenic dyslipidemia, i.e., patients with TG levels beyond 2.26 mmol/l (200 mg/dl) and HDL-cholesterol levels below 0.9 mmol/l (35 mg/dl) are likely to derive the greatest CV protection from the addition of IPE to their regular statin-based therapy [52]. Importantly, routine screening for high CV risk and risk enhancers remains suboptimal in clinical practice; however, this inertia invariably leads to significant residual CV risk. In fact, guidelines recommend identification of the different CV risk factors and the stratification of patients to inform best therapeutic decisions. Therefore, every patient fulfilling any of the below CV risk enhancers must be evaluated for additional lipid-lowering therapy in order to target residual CV risk. As a rule of thumb, clinical care starts by aggressively targeting LDL-cholesterol with statins and then adding ezetemibe, PCSK9 inhibitors, or IPE to patients who achieved controlled LDL-cholesterol levels or to those with statin resistance or intolerance. Patients with severe mixed dyslipidemia should invariably be on the highest tolerated statin dose, which should be optimized before starting IPE. Patients with severe hypertriglyceridemia are also at risk of pancreatitis, and these patients can be prescribed IPE on top of statins, and then as needed, fibrates.

### Are the Safety Events Reported with IPE Use Clinically Relevant in the Context of CV Risk Reduction?

The net clinical benefit of IPE was sustained in all treated population subgroups and far outweighs potential risks. In fact, while the proportion of any bleeding was higher (*P* = 0.006) in the IPE group (11.8%) compared to placebo (9.9%)), the rate of hemorrhagic stroke did not reach statistical significance (*P* = 0.54), while serious bleeding events were more frequent in the IPE group (2.7% [111/4089] versus 2.1% [85/4090] in the placebo group, *P* = 0.06) . In addition, the frequency of bleeding events in aspirin-treated patients is higher than that reported upon IPE use [113]. More atrial fibrillation/flutter events were recorded in the IPE group (*P* = 0.002), including serious ones requiring hospitalization longer than 24 h (*P* = 0.008) [52]. However, major CV events, including stroke, were still reduced in the IPE arm, and some patients already had a history of atrial fibrillation at baseline. Indeed, recurrent atrial fibrillation/flutter was more frequent than de novo atrial fibrillation/flutter and those patients especially experienced marked CV benefits upon IPE use [114]. A subgroup analysis of REDUCE-IT showed that rates of hospitalization for atrial fibrillation were higher in patients with prior atrial fibrillation (12.5% in the IPE versus 6.3% in the placebo group, *P* = 0.007), compared to patients without history of atrial fibrillation (2.2% versus 1.6%, respectively, *P* = 0.09) [115].

Overall, fewer CV events occurred in the IPE arm, compared to placebo [113] and, in particular, the risk of an increase in hospitalization due to atrial fibrillation/flutter and the trend toward increased serious bleeding risk with IPE are offset by its CV benefits [114, 115]. In addition, recommendation for IPE use by international scientific and medical associations attests to the fact that its potential benefits greatly outweigh the safety concerns that should, nevertheless, continue to be monitored in clinical practice.

### How to Leverage Residual CV Risk Surveillance and Management at the Primary Care Level?

While CVD should be diagnosed and managed at secondary or tertiary care centers, by a multidisciplinary team of experienced cardiologists and other specialties, primary care remains important for the identification of CV risk factors and early signs of CVD in the majority of the population with no prior CV event. In fact, “red flags” can be picked up by any physician, and screening for *risk enhancers* must be routinely performed in primary care settings. Risk enhancers include family history of atherosclerotic CVD, metabolic syndrome, persistently elevated LDL-cholesterol, low HDL-cholesterol, elevated TG levels, high hsCRP levels, chronic kidney disease, chronic inflammatory diseases, etc. [116]. Patients eligible for lipid-lowering treatment can be evaluated for eligibility for IPE in primary or secondary prevention; hence the need to train primary care physicians and nurses to evaluate patients, according to criteria listed in Table 2. Even patients with varying (elevated or modestly elevated; i.e., TG > 1.69 mmol/l or 150 mg/dl) TG levels can benefit from IPE. From a surveillance standpoint, patients from increased risk groups and patients with modest elevation of TG will be provided with greater CV protection with IPE; which should be prescribed at the primary care level. The following URL (https://www.acc.org/~/media/Non-Clinical/Files-PDFs-Excel-MS-Word-etc/Guidelines/2018/Guidelines-Made-Simple-Tool-2018-Cholesterol.pdf) links to a document that can serve to direct screening and management of CV risk-enhancing factors at primary care settings.

### To What Extent Does Health Hygiene (Including Fish-Rich Diet) impact CVD Onset, Progression or Regression in Patients at High CV Risk or with Residual CV Risk?

Unhealthful behaviors predispose individuals to a plethora of health conditions, underscoring the importance of primordial prevention in the general population or primary prevention in the population at risk for certain diseases. In particular, CVD are largely preventable if underlying risk factors are addressed in a timely manner. Regular physical activity, abstinence from smoking, low-fat and low-sugar diets, and moderate alcohol consumption and avoidance of pollutants are of utmost importance in preventing CVD or in mitigating symptoms and delaying complications. It is widely accepted that populations with high fatty fish intake and with elevated serum omega-3 fatty acid levels have traditionally lower CV risk and greater longevity [117, 118]. Despite the epidemiological data offering a glimpse of hope for the prevention of CVD and outcome exacerbations [119, 120], trials exploring omega-3 fatty acid-rich fish oil supplementation have failed to present evidence on improved CV outcomes in patients with CVD [78, 92, 93, 121]. This limited CV benefit of omega-3 fatty acids can be attributed to changes in their physical properties, but also to the fact that patients in these trials all had established CVD. Although nutrition and population-based studies underscore the importance of omega-3 fatty acids, therapeutic levels of serum EPA are difficult to attain from a sporadic omega-3-rich diet; coupled with the impracticality of motivating dietary habit change in populations with specific cultural and socio-economic underpinnings.

### In Terms of Health Economics, what are the Benefits of Using IPE to Further Mitigate CV Risk in Statin-Treated Patients?

Some people with limited financial resources might turn to free or cheaper treatment alternatives to avoid paying charges, but this approach might result in higher rather than lower healthcare expenditure [122]. Rosuvastatin alone or with candesartan and hydrochlorothiazide proved a cost-saving prescription in higher income countries, but resulted in higher expenditure on health in developing countries [123]. With particular interest in dyslipidemia management, treatment with PCSK9 inhibitors to decrease levels of LDL-cholesterol was reported in 2016 to be too expensive and unaffordable by health systems [124] and in 2018, the American College of Cardiology guidelines concluded that the price of PCSK9 inhibitors must be reduced by almost 70% to meet cost-effectiveness standards [125]; although a more recent study has proven PCSK9 inhibitors to be cost-effective only at a substantially lower cost and in the very-high-risk population [126]. A very recent study evaluated the cost-effectiveness of adding IPE treatment to statin treatment for CV risk reduction and found that it would be especially cost-effective in the context of secondary prevention of CVD [127]. Importantly, REDUCE-IT shows that CV event frequency decreases upon IPE treatment [80], which inherently improves clinical outcomes and decreases the use of healthcare resources. In the United States, IPE was found to be cost-effective in primary prevention and associated with even better outcomes at lower cost for secondary prevention purposes [128]. The clinical need is undeniable, provided the acquisition cost of IPE fits with the budget of stakeholders involved in medication provision and coverage (government subsidies, third-party payers, out-of-pocket expenditure, etc.). Patients and physicians alike should be made aware that the higher cost of IPE is offset by its effectiveness in preventing CVD, in reducing the recurrence of CV events that require medical care, and in mitigating overall mortality and CV burden.

## Conclusion

Elevated TG levels are not only a biomarker of CV risk, but also a mediator of CVD, conferring a significant residual CV risk persisting in patients treated with lipid-lowering agents. Fundamentally, even modestly elevated TG levels are a risk factor that can be very effectively managed with a specific agent (IPE) that has very robust evidence in reducing CV risk. Mixed omega-3 fatty acid formulations have been shown to decrease TG levels, without, however, eradicating the residual CV risk. IPE, through the robust and unequivocally positive REDUCE-IT results, revolutionized the use of pure omega-3 fatty acids in primary and secondary prevention of CVD. IPE showed marked and sustained CV benefits in patients with high TG and low HDL-cholesterol levels, but also in patients presenting with comorbid conditions, known as CV risk enhancers, metabolic syndrome and atherogenic dyslipidemia, which are particularly prevalent in some populations such as in the Middle East. IPE was indeed evaluated in different population subgroups, which are clinically relevant in daily practice; as reflected in the most updated guidelines. IPE seems to exert its multiple CV protective effects through the attenuation of the inflammatory response, the stabilization of the plasma membrane, the regression of atherosclerosis and, ultimately, the reinstatement of normal vascular function. IPE is also generally well tolerated, despite a higher risk of bleeding and atrial fibrillation, which did not compromise the strong clinical net benefit of IPE. Since its approval by the Food and Drug Administration in 2019 and then by the European Medicines Agency in 2021, IPE has shifted the paradigm of hypertriglyceridemia management in clinical practice [129]. The rather broad benefits conferred by IPE make it cost-effective at a societal level, which should prompt its rapid adoption as a pillar of treatment for both dyslipidemia and CV risk.

## Data Availability

Not applicable.
